# An acute Q fever with vessel vasculitis: case report

**DOI:** 10.3389/fmed.2025.1584216

**Published:** 2025-07-11

**Authors:** Shijun Guo, Quanle Liu, Jiaqi Yan, Jin Wang, Yingran Zhong, Feng Gao, Yuejia Zhong

**Affiliations:** ^1^Guangdong Provincial Hospital of Chinese Medicine, The Second Affiliated Hospital, Guangzhou University of Chinese Medicine, Guangzhou, China; ^2^Second Clinical Medical College, Guangzhou University of Chinese Medicine, Guangzhou, Guangdong, China; ^3^Yuexiu District Hospital of Chinese Medicine, Guangzhou, China

**Keywords:** Q fever, vessel vasculitis, case report, NGS, PET/CT

## Abstract

**Background:**

Q fever (QF) is a relatively rare zoonotic infectious disease, and complications such as vasculitis and endocarditis are uncommon but severe. This article reports a case of acute QF complicated by vasculitis.

**Case presentation:**

The patient presented with a week of recurrent fever. Upon admission, inflammatory markers and liver transaminases were elevated, and a weak positive result for Chlamydia pneumoniae Immunoglobulin M (IgM) antibodies was detected. After treatment with levofloxacin and doxycycline, the fever persisted. Blood metagenomic next-generation sequencing (mNGS) suggested *Coxiella* species, raising suspicion for acute QF. The antibiotics were switched to moxifloxacin, but fever continued. Autoimmune tests showed positive antinuclear antibodies, and multiple blood cultures were negative. Further positron emission tomography/computed tomography (PET/CT) revealed inflammatory changes at the bifurcation of the right internal and external carotid arteries, as well as the ascending aorta, pulmonary arteries, and descending aorta, suggesting QF complicated by vasculitis. Treatment with methylprednisolone led to gradual resolution of the fever, and rechecked autoimmune antibodies turned negative. The patient did not experience further fever after discharge.

**Conclusion:**

Currently, early recognition and diagnostic techniques for QF still require further improvement. As an infectious disease, timely treatment and vaccination for QF remain key areas of focus for future healthcare professionals.

## Background

Q fever (QF) is a zoonotic infectious disease caused by *Coxiella burnetii* infection (1). The primary mode of transmission is the inhalation of aerosolized particles containing the pathogen, with domestic animals, wildlife, and ticks being the main hosts (2). Clinically, QF commonly presents with fever, headache, and muscle pain, but lacks specificity. The fever typically lasts for 1–3 weeks. Common sites of infection include the lungs, liver, and endocardium, and it can lead to multi-organ failure (3). This article reports a case of acute QF complicated by vasculitis.

## Case presentation

A 66-year-old male was admitted on January 15, 2022, due to recurrent fever for 1 week, presenting with chills, fatigue, and a fluctuating body temperature between 37.5°C and 38.5°C, with higher fever in the afternoon. He also experienced headache, dizziness, and diarrhea for 1 day, with 10 episodes of loose, foul-smelling stools. His vital signs were measured as follows: temperature 36.6°C, heart rate 108 beats/min, respiratory rate 20 breaths/min, and blood pressure 165/91 mmHg. Physical examination revealed normal cardiopulmonary and abdominal findings. He had no oral ulcers, joint pain, cough, sputum production, abdominal pain, diarrhea, or urinary symptoms such as frequency, urgency, or dysuria. Upon inquiring about his medical history, we learned that he had a history of asthma, gallstones, and right parotid gland surgery. He works as an engraver and lives at the foot of Baiyun Mountain in Guangzhou, where he has frequent contact with birds.

After admission, we conducted relevant tests. The results were as follows: complete blood count showed white blood cells at 7.38 × 10^∧^9/L, with neutrophils making up 71.2%; procalcitonin was 1.96 ng/L; alanine aminotransferase (ALT) was 116 U/L; aspartate aminotransferase (AST) was 92 U/L; total bilirubin was 12.4 μmol/L; direct bilirubin was 4.0 μmol/L; coagulation tests showed fibrinogen at 4.17 g/L, and D-dimer was 2.8 mg/L. Chest computed tomography (CT) did not show any abnormalities. Abdominal CT indicated mild fatty liver and gallstones with accompanying cholecystitis. Respiratory viral antigen testing showed weakly positive Immunoglobulin M(IgM) antibodies for *Chlamydia pneumoniae*, while Q fever rickettsia IgM antibodies were negative.

Therefore, based on the clinical evidence, both cholecystitis and atypical pneumonia are considered possible diagnoses, though the causative pathogen remains unidentified. An empirical antibiotic therapy has been initiated accordingly. Therefore, for treatment, we administered levofloxacin 0.5 g once daily via intravenous drip for infection control and added doxycycline 0.1 g every 12 h orally on January 17 to cover atypical pathogens. Despite this, the patient continued to experience recurrent fever. On January 19, we performed metagenomic next-generation sequencing (mNGS, Table 1), which suggested *Coxiella* species with a sequence count of 540, leading to a diagnosis of acute Q fever. Consequently, we switched the antibiotic from levofloxacin to moxifloxacin 0.4 g every day intravenously for infection management.

**TABLE 1:** mNGS of the patient.

Date	Type	Genus		Species	
		**Latin name**	**Number of detected sequences**	**Latin name**	**Number of detected sequences**
2022.1.19	G^–^	*Coxiella*	540	*Coxiella burnetii*	540
2022.1.30	Not found				

Despite our active antimicrobial treatment, the patient continued to experience daily fever, with body temperature fluctuating between 36°C and 40.4°C. A follow-up chest X-ray on January 24 showed bilateral lower lung inflammation with a small amount of pleural effusion. During hospitalization, the antibiotic regimen was as follows: levofloxacin 0.5g once daily via intravenous drip (01.15–01.18), doxycycline 0.1g every 12 h orally (01.17–02.01), and moxifloxacin 0.4g once daily via intravenous drip (01.19–02.01). Bone marrow aspiration showing reactive proliferative changes in the bone marrow suggests that inflammation persists. A follow-up blood mNGS on January 30 (Table 1) was negative. Transesophageal echocardiography revealed no vegetation, ruling out infective endocarditis. Autoimmune tests for 16 items showed positive antinuclear antibodies (1:100 titer), ribosomal P protein antibodies 129 AU/ml, anti-SSB antibodies 115 AU/ml, anti-Jo-1 antibodies 148 AU/ml, and anti-nucleolar antigen antibodies 162 AU/ml, with the remaining items negative.

**FIGURE 1:**
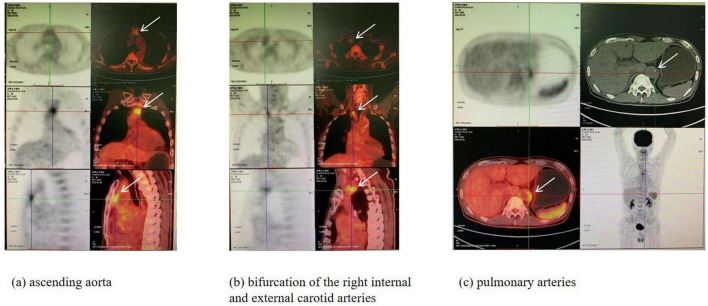
PET/CT of the patient (2022.02.04). Multiple nodular and strip-shaped hypermetabolic lesions were observed at the bifurcation of the right internal and external carotid arteries, as well as in the ascending aorta, pulmonary arteries, and descending aorta. There were also strip-shaped and nodular hypermetabolic lesions in the pericardium. **(a)** The arrows indicate hypermetabolic foci in the ascending aorta. **(b)** The arrows point to hypermetabolic foci at the bifurcation of the right internal and external carotid arteries. **(c)** The arrows highlight hypermetabolic foci in the pulmonary arteries.

The patient continued to experience recurrent fever, and multiple blood cultures remained negative. The cause of fever remains undetermined. On February 4, a positron emission tomography/computedtomography (PET/CT) scan was performed, which revealed inflammatory changes at the bifurcation of the right internal and external carotid arteries, as well as the ascending aorta, pulmonary arteries, and descending aorta, suggesting QF complicated by vasculitis (Figure 1). Therefore, we started treatment with methylprednisolone 40mg once daily via intravenous drip (02.09–02.14). Later, autoimmune tests for 16 items showed positive antinuclear antibodies (1:100 titer), with all other items negative. After steroid treatment, the fever subsided, and the patient was stable. Re-examination of inflammatory markers and liver function tests on February 9 all showed normal results (white blood cells was 10.38 × 10^∧^9/L; procalcitonin was 0.09 ng/L; ALT was 18 U/L; AST was 16 U/L.). The patient’s condition remained stable and was discharged on February 14. A follow-up on February 15 showed positive QF rickettsia IgM antibodies in respiratory viral testing. The patient had no further fever after discharge. During the 2-week follow-up period, no fever or other discomfort symptoms were observed.

## Discussion

In this case, several aspects need to be considered: first, the etiological and pathological mechanisms of QF complicated by vasculitis and its identification; second, the microbiological diagnostic methods for QF to ensure accurate recognition and timely diagnosis; and third, the treatment and preventive measures for QF complicated by vasculitis.

Currently, there are few case reports worldwide regarding QF complicated by vasculitis, and the related mechanisms remain unclear. *Coxiella burnetii* is a strictly intracellular bacterium and the pathogen responsible for QF, which is a zoonotic disease. It is commonly found in the secretions or excretions of domestic animals such as cattle, sheep, and poultry, as well as in wild rodents and migratory birds, which are also highly infectious (4). Ticks serve as both the parasitic host and the reservoir host, and they are vectors for transmission between animals. The disease is typically transmitted via the respiratory and gastrointestinal tracts (5). Humans can contract the infection by inhaling aerosolized particles containing the pathogen, with even low doses being sufficient to cause infection. Common symptoms include fever, muscle aches, and elevated transaminases.

*Coxiella burnetii* enters host cells through internalization and reproduces within vesicles formed by phagosomes. It then enters the bloodstream via cell lysis, which can lead to diseases affecting multiple systems (6). Studies have shown that *Coxiella burnetii* can proliferate extensively in arterial endothelial cells and, in severe cases, cause arterial occlusion (7). Additionally, a nationwide survey in France found that *Coxiella* infection can induce small-vessel mixed cryoglobulinemic vasculitis, medium-vessel vasculitis, and granulomatous large-vessel vasculitis (7), causing varying degrees of damage to the systemic vasculature. PET/CT scans are mainly used to assess vascular involvement in QF patients (8). Chronic *Coxiella* infection typically involves localized infection, with common sites including the vasculature and endocardium. A study involving 40 QF patients found that 32 of them had aortic inflammation, indicating that the aorta is one of the common sites of infection in QF (9). In this case, PET/CT confirmed aortic inflammatory changes. Furthermore, the autoimmune antibodies produced in the blood are often associated with chronic *Coxiella* infection (10). The patient’s first autoimmune test for 16 items revealed multiple positive autoimmune antibodies, but after treatment, many of these antibodies turned negative, indirectly supporting this association.

The incubation period for QF is approximately 2–3 weeks (11). Therefore, the *Coxiella burnetii* IgM antibodies were negative when the patient presented, which posed a challenge for early identification and diagnosis. Since *Coxiella burnetii* is an intracellular bacterium, blood cultures are typically negative. So the definitive diagnosis of the pathogen often relies on nucleic acid and serological tests (12). Commonly used serological methods include the complement fixation test (CFT), enzyme-linked immunosorbent assay (EIA), and immunofluorescence assay (IFA), with IFA being the gold standard for detecting *Coxiella burnetii* antibodies (13). However, in China, testing for phase I and phase II *Coxiella* antibodies has not yet been implemented, and currently, Q fever rickettsia IgM is the available serological test. IgM antibodies are the first antibodies secreted during the immune response (14) and can be produced quickly after infection, with a positive result often indicating recent infection. IgM typically becomes negative within 2–4 weeks. Previously, diagnosing QF posed challenges, including delays in results and low positivity rates. In recent years, mNGS has become a new tool for the rapid detection of infectious disease pathogens, significantly improving the diagnostic rate for some hidden infectious diseases (13, 15). PET/CT is also one of the diagnostic methods for unexplained fever (16). In this case, aortic inflammation was subtle and was ultimately diagnosed through PET/CT. In 2022, a case (17) was reported involving a febrile patient from China, who remained undiagnosed and untreated for 3 weeks until mNGS detected *Coxiella burnetii* genomic sequences in the patient’s blood. This case further underscores the critical role of diagnostic methods and clinicians’ awareness in timely disease management. Although diagnostic methods for the pathogen have increased, the diagnosis of QF still requires comprehensive analysis of epidemiological history, clinical symptoms, and serological testing. As the disease can affect multiple systems and involves cross-disciplinary concerns, this emphasizes the importance of multidisciplinary consultations when necessary.

The first-line treatment for QF is antimicrobial therapy, with tetracyclines being the preferred class. Doxycycline 100mg twice daily is commonly used, with a treatment course typically lasting 2 weeks. For patients with vasculitis, corticosteroid therapy can achieve good therapeutic effects. In this case, after 6 days of treatment with methylprednisolone, the patient’s fever gradually subsided, and no further fever was observed after discharge, resulting in a satisfactory treatment outcome. However, for infectious diseases, prevention is better than treatment. Vaccination is the most effective preventive measure (18). Currently, research and development of vaccines for QF are increasing, but their safety still needs further investigation. As studies on *Coxiella* species-specific responses progress both domestically and internationally, the availability of a QF vaccine is anticipated in the future.

There are some limits in the report. In this case, the diagnosis was solely based on IgM antibody detection for *Coxiella burnetii* using IFA, without performing antibody titer determination. Additionally, since China currently lacks testing capabilities for *Coxiella burnetii* phase I and phase II antibodies, this has somewhat limited our understanding of the patient’s immune response. Additionally, the patient was only followed up for 2 weeks after discharge. The short follow-up period precludes assessment of long-term outcomes or late adverse effects. Publication bias may exist as this case was selected due to its unusual presentation, which may not reflect typical clinical scenarios.

## Conclusion

Although the incidence of QF is relatively low and most patients have a good prognosis, serious complications such as vasculitis and endocarditis should not be overlooked. Early identification through serological testing and clinical symptoms, along with timely intervention, is crucial. At the same time, as an infectious disease, the prevention and treatment of QF remain key areas for future research.

## Data Availability

The original contributions presented in this study are included in this article/supplementary material, further inquiries can be directed to the corresponding author.
